# A Rare Cause of Chronic Hip Pain From PEComa: An Aggressive Mesenchymal Sarcoma

**DOI:** 10.1177/23247096221103385

**Published:** 2022-06-14

**Authors:** Samender Randhawa, Jessica Pinsker, Madhurya Amirapu, Metlapalli Venkata Sravanthi, Prashanth Ashok Kumar, Komal Akhtar

**Affiliations:** 1 State University of New York Upstate Medical University, Syracuse, USA

**Keywords:** PEComa, sarcoma, epithelioid, tumor, hip pain, mesenchymal, metastatic, TFE3, sirolimus

## Abstract

Perivascular epithelioid cell tumors (PEComas) are related to the tuberous sclerosis complex (TSC) and are commonly benign. When malignant, they can be aggressive with local invasion and metastatic spread. Conventional PEComas do not have *TFE3* gene rearrangement and are associated with TSC with a preference for an occurrence at a younger age. We report a case of a young male who had progressive chronic hip pain and was found to have a *TFE3*-associated PEComa in his pelvic region.

## Background

Perivascular epithelioid cell tumors (PEComas) are a family of rare tumors showing perivascular epithelioid cell differentiation that originates in the soft tissues of any organ in the body. The most common site of origin is in the abdominopelvic region, gastrointestinal tract, retroperitoneum, and uterus.^1,2^ Most PEComas are often related to the tuberous sclerosis complex (TSC).^1^ They are mostly benign and curable by surgical resection when benign.^2^ Here, we describe a case of a young male who had progressive chronic hip pain that was found to have a PEComa associated with *TFE3* translocation in his pelvic region. We also describe a rare subset of PEComas that has an absence of association with TSC and has a striking nuclear positivity for *TFE3.*^3^

## Case Presentation

The patient is a 33-year-old Caucasian male who presented after a motor vehicle accident and was found to have multiple fractures from pelvic trauma. On imaging, the patient was incidentally found to have a large pelvic mass. The patient underwent embolization for internal bleeding along with placement of hardware in the pelvic region to address the fractures. Postoperatively, the patient mentioned experiencing chronic right hip pain for the last 2 years. Since the pain did not functionally limit, immediate medical attention was not sought early.

**Figure 1.**
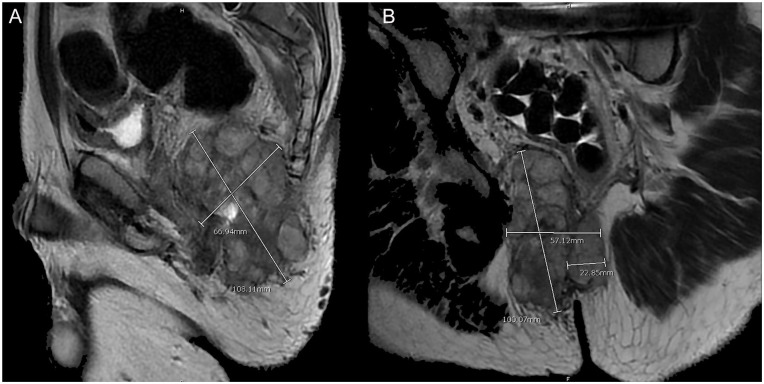
(A) MRI of the pelvis showing the soft tissue mass in the sagittal view. (B) MRI of the pelvis showing the soft tissue mass in the coronal view. Abbreviation: MRI, magnetic resonance imaging.

**Figure 2.**
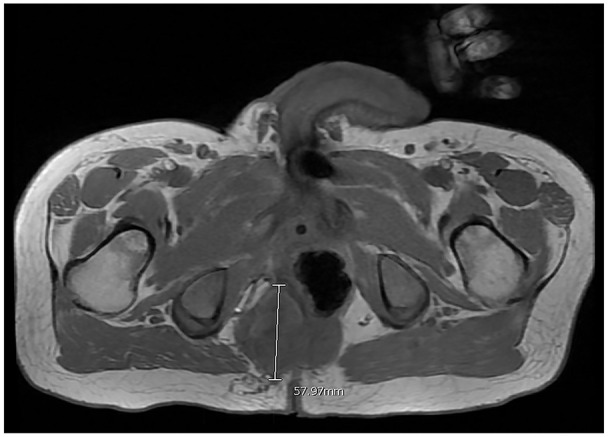
Magnetic resonance imaging (MRI) of the pelvis showing the axial view of the soft tissue mass.

The magnetic resonance imaging (MRI) of the pelvis showed a large soft tissue mass centered within the right ischiorectal fossa that measured 6.6 cm × 6.9 cm × 10.8 cm. Inferiorly, the mass extended 2.3 cm across the midline with displacement of the rectum toward the left (Figures 1 and 2).

The biopsy of the pelvic mass showed a smooth muscle tumor with epithelioid morphology. The pathology showed the tumor to consist of cohesive clusters of vague nests of epithelioid cells with eosinophilic cytoplasm, which was somewhat granular, and had uniform vesicular nuclei. Immunostaining the tissue biopsy showed striking positivity for desmin, while SMA, caldesmon, HMB45, Melan-A, microphthalmia-associated transcription factor (MITF), and MYOD1 were negative. The granular character of the cytoplasm favored the diagnosis of PEComa. There was striking nuclear positivity for *TFE3*. Although *TFE3* is not truly specific, in the context of the morphology and desmin positivity, the pathologic appearance was consistent with PEComa, likely associated with *TFE3* gene rearrangement. The Ki-67 proliferation index was 15.3% and tissue necrosis was visible in many parts of the biopsy.

**Figure 3.**
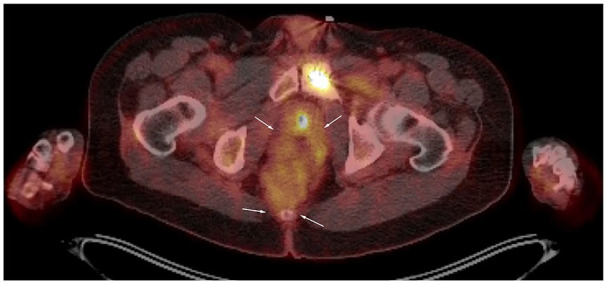
Positron emission tomography (PET)/computed tomography (CT) scan showing the perirectal perivascular epithelioid cell tumor (PEComa), outlined with arrows.

After discussion with both radiation oncology and orthopedic surgery, a shared decision was made with the patient to start neoadjuvant chemotherapy. He was started on doxorubicin, ifosfamide, and mesna with the intent of decreasing the size of the mass in anticipation of surgical resection. A positron emission tomography (PET)/computed tomography (CT) after 2 cycles demonstrated a mild decrease in the size of the mass, which measured approximately 6.7 cm × 5.7 cm (Figure 3). No distant metastasis was noted.

The plan moving forward would be to consider surgical resection once sufficient tumor shrinkage is achieved.

## Discussion

The most common form of PEComas is angiomyolipoma (AML), specifically renal AML. Other forms include lymphangioleiomyomatosis (LAM) and clear cell “sugar” tumor of the lung.^4,5^ Perivascular epithelioid cell tumors may contain epithelioid or spindle-shaped cells while their cytoplasm can range from being clear to eosinophilic.^4^ Nearly all PEComas show immunoreactivity for both melanocytic (HMB-45 and/or melan-A) and smooth muscle markers.

The criteria currently used for the malignant classification of PEComas include tumor size >5 cm, infiltration, nuclear grade, cellularity, necrosis, vascular invasion, and mitotic rate as important prognostic factors.^2,6^ The size of the tumor in our patient along with the histopathologic characteristics provides a potential for malignant spread. However, cellularity and the mitotic rate were not calculated because the tissue biopsy sample was not completely viable due to the preoperative embolization. Nevertheless, based on the size, possibility of true necrosis on biopsy, and the high proliferation index of the tumor, the patient was treated for a potential malignant PEComa with neoadjuvant chemotherapy with plan for surgical resection in the future.

Conventional PEComas are typically associated with TSC in young patients, but also frequently have a spindle cell component. They stain positive for muscle markers such as actin and desmin and lack strong *TFE3* immunoreactivity. In contrast, there is a subset of lesions classified as PEComas but harbor *TFE3* gene fusions. Distinctive features of *TFE3*-associated PEComas include a tendency to occur at a younger age, no association with TSC, minimal immunoreactivity for actin or desmin markers, predominant epithelioid cytology, and strong *TFE3* immunoreactivity.^7^ *TFE3*-associated PEComas are mutually exclusive to those associated with TSC.^5^ It has been suggested that PEComas that express *TFE3* immunoreactivity but do not involve the *TSC2* gene may be biologically distinctive from conventional PEComas. These patients may not benefit from mammalian target of rapamycin (mTOR) inhibitors.^4^ One study found *TFE3* rearrangements in 23% of all diagnosed PEComa cases.^8^ Our case represents one such nonconventional PEComa. This case differed from other reported *TFE3*-rearranged PEComas in that immunostaining showed positivity for desmin and *TFE3*. Our patient did not have any clinical features or known family history of TSC.

Most PEComas are benign and occur in association with TSC. In TSC, the genes that undergo mutations are *TSC1* on chromosome 9q34 and *TSC2* on chromosome 16p13.3, which serve to regulate cell division and differentiation.^5^ In conventional PEComas, there is a loss of *TSC2.*^4^ Inactivation of *TSC1* and *TSC2* genes, seen in TSC as well as in sporadic PEComas, is associated with subsequent activation of the mTOR pathway. Therefore, treatment with mTOR inhibitors such as sirolimus (ABI-009, previously called rapamycin) has shown a clinical response in a number of patients with AML, LAM, malignant PEComas, and other TSC-related lesions.^5^ However, radical resection is still the primary treatment for PEComas.^2^

In the recently published phase II AMPECT (ABI-009 in Patients With Advanced Malignant PEComa) clinical trial, 31 patients treated with nab-sirolimus 100 mg/m^2^ showed an overall response rate (ORR) of 39%.^9^ While 1 patient had a complete response, 52% had stable disease. This represented an important alternative treatment option for patients with malignant PEComas and was approved by Food and Drug Administration (FDA) based on this trial.^10^ Traditional doxorubicin-based chemotherapeutic regimes have shown response rates <20%.^11^ Comparative trials remain elusive due to the uncommon nature of the disease.

The overall clinicopathologic features of the nonconventional subset of PEComas remain poorly understood due to their extreme rarity. The treatment for malignant PEComas is still not defined and requires further studies. Closer follow-up of patients with nonconventional PEComas as they undergo treatment will provide much-needed insight and prognostic information for the development of future protocols.
